# Landscape effects and spatial patterns of avian influenza virus in Danish wild birds, 2006–2020

**DOI:** 10.1111/tbed.14040

**Published:** 2021-05-06

**Authors:** Lene Jung Kjær, Charlotte Kristiane Hjulsager, Lars Erik Larsen, Anette Ella Boklund, Tariq Halasa, Michael P. Ward, Carsten Thure Kirkeby

**Affiliations:** ^1^ Faculty of Health and Medical Sciences Section for Animal Welfare and Disease Control Department of Veterinary and Animal Sciences University of Copenhagen Frederiksberg Denmark; ^2^ Department of Virus & Microbiological Special Diagnostics Statens Serum Institut Copenhagen Denmark; ^3^ Faculty of Science Sydney School of Veterinary Science The University of Sydney Camden NSW Australia

**Keywords:** AIV surveillance, Avian influenza, high‐risk clusters, landscape, spatial patterns, wild birds

## Abstract

Avian influenza (AI) is a contagious disease of birds with zoonotic potential. AI virus (AIV) can infect most bird species, but clinical signs and mortality vary. Assessing the distribution and factors affecting AI presence can direct targeted surveillance to areas at risk of disease outbreaks, or help identify disease hotspots or areas with inadequate surveillance. Using virus surveillance data from passive and active AIV wild bird surveillance, 2006−2020, we investigated the association between the presence of AIV and a range of landscape factors and game bird release. Furthermore, we assessed potential bias in the passive AIV surveillance data submitted by the public, via factors related to public accessibility. Lastly, we tested the AIV data for possible hot‐ and cold spots within Denmark. The passive surveillance data was biased regarding accessibility to areas (distance to roads, cities and coast) compared to random locations within Denmark. For both the passive and active AIV surveillance data, we found significant (*p* < .01) associations with variables related to coast, wetlands and cities, but not game bird release. We used these variables to predict the risk of AIV presence throughout Denmark, and found high‐risk areas concentrated along the coast and fjords. For both passive and active surveillance data, low‐risk clusters were mainly seen in Jutland and northern Zealand, whereas high‐risk clusters were found in Jutland, Zealand, Funen and the southern Isles such as Lolland and Falster. Our results suggest that landscape affects AIV presence, as coastal areas and wetlands attract waterfowl and migrating birds and therefore might increase the potential for AIV transmission. Our findings have enabled us to create risk maps of AIV presence in wild birds and pinpoint high‐risk clusters within Denmark. This will aid targeted surveillance efforts within Denmark and potentially aid in planning the location of future poultry farms.

## 1 INTRODUCTION

Avian influenza (AI) is a contagious disease of birds with zoonotic potential. It is caused by Influenza A viruses (AIV), and can be classified as low pathogenic (LPAI) and high pathogenic (HPAI) subtypes based on their pathogenic phenotype. Only AIV of subtype H5 and H7 are known in the HPAI form. LPAI is a persistent problem worldwide. LPAI is found in most bird species, and LPAI subtypes H5 and H7 have the potential to mutate into HPAI, which can cause great economic loss and animal welfare problems when farmed birds are infected (Monne et al., 2014; Rao et al., 2009). Furthermore, some AIV subtypes have zoonotic potential with high case‐fatality for humans (Lai et al., 2016); thus, it is crucial to monitor and prevent the geographical spread of AIV in both wild and farmed birds. Control measures to prevent the dispersal of AIV include transport restrictions between areas at risk, contact tracing, hygiene measures and culling exposed animals (Stegeman et al., 2004).

Several countries have implemented surveillance programmes to monitor the distribution of AI and evaluate the spatio‐temporal risk, both for wild and farmed birds (Bevins et al., 2014; Buscaglia et al., 2007; Hesterberg et al., 2009; Machalaba et al., 2015). Data obtained from these surveillance programmes can aid in developing statistical spatio‐temporal models to identify high‐risk areas and critical time periods, which can optimise surveillance for AI. Prediction models for AIV occurrence and risk of HPAI outbreaks have, to a large extent, focused on landscape use, which can indicate the density of specific birds with higher risk of transmitting AIV (Gilbert et al., 2008; Paul et al., 2010; Ward, Maftei, Apostu, & Suru, 2008, 2009). Studies have also described continental hotspots for AIV subtypes (Bevins et al., 2014), showing that it is possible to identify risk factors on a large geographical scale. Denmark has dense wild birding areas that intersect with many bird migration routes, including routes coming to and from Europe (Bregnballe et al., 1997), Africa (Tøttrup et al., 2018) and Siberia (Dick et al., 1987). Therefore, there is a high potential for AIV incursions from other regions. In particular, migratory birds from Siberia appear to be a risk factor, as Siberia has previously been identified as a major hub for AIV dispersal (Lai et al., 2016; Li et al., 2014). Additionally, a large number of game birds are released every year for hunting in Denmark (Gamborg et al., 2016; Kanstrup et al., 2009). Some of these game birds originate from other countries (Ministry of Environment & Food of Denmark, 2020; The Danish Hunting Association, 2020), increasing the potential of introducing AIV into Denmark.

Since 2002, the Danish authorities have carried out surveillance for AIV in wild birds. We obtained data from this surveillance system generated between 2006 and 2020 and explored potential patterns of AIV occurrence and spatial risk factors in Denmark. The aim of the study was to identify areas with high or low occurrence of AIV and possible factors associated with these occurrences, in order to optimize future surveillance for AIV. Furthermore, we aimed to assess bias in the Danish passive AIV surveillance data submitted by the public by assessing variables related to human accessibility. Potentially, our results can be applied to future planning efforts; for example, high‐risk areas should be excluded when planning the location of future poultry farms.

## 2 MATERIALS AND METHODS

### 2.1 Passive and active AIV surveillance data

We obtained virus detection data from both passive (2006−2020) and active (2007−2019) wild bird AIV surveillance. Passive surveillance data were from the EU mandatory passive surveillance program in Denmark, in which dead and diseased wild birds are tested for AIV and particularly H5/H7 subtypes, whereas the active surveillance data are based on samples from healthy birds, captured for sampling or ringing, submitted by hunters, or from bird dropping samples. Some of the observations in the active AIV surveillance data were pooled samples, whereas others were from individual birds, which had to be taken into account when analysing the data (Section 2.4).

All data were manually checked for entry errors and plotted in ArcMap 10.6.1 (Environmental Systems Research Institute, 2017) to check for any errors in the coordinates (such as coordinates not being located within Denmark). The passive location data all had UTM coordinates for where the birds were found. As the birds from the passive AIV surveillance data were found by the public, we suspected it to be biased due to varying detection probabilities as well as human accessibility to wildlife areas. To assess this, we compared various accessibility variables of the passive AIV surveillance location data to random locations within Denmark (section 2.3 and 2.4). The active surveillance data only had precise UTM‐coordinates from 2007 to 2010. From 2011 to 2019 the active surveillance data only registered the postal code of where the sample was collected. To create one single dataset for the active surveillance data, we converted the 2007−2010 coordinates to postal codes instead, and conducted all our analyses on active surveillance data at the postal code level. We also created a single wild bird AIV surveillance dataset by combining the passive and active AIV surveillance data, leaving us with three datasets on which to conduct our analyses—the passive AIV surveillance data, the active AIV surveillance data, and a combined wild bird AIV surveillance dataset. When combining the active and passive AIV surveillance data, we converted all the passive surveillance data coordinates to postal codes, producing a combined dataset based on postal codes alone. We refer to this combined dataset as the wild bird AIV surveillance data throughout this paper.

### 2.2 Data on game birds

We obtained data from 2018 to 2019 on game birds bred and released for hunting from the Danish.

Environmental Protection Agency. This data had addresses only and no coordinates, thus we used ArcGIS World Geocoding Service (Environmental Systems Research Institute, 2017) to transform all addresses to UTM coordinates. In some cases, only a postal code was reported for the release site, and not a complete address. In those cases, we used the centroid coordinates of the total area of that particular postal code. These centroid coordinates were obtained from a shape file of all Danish postal codes and their areas (Danish Map Supply; Kortforsyningen, 2020). There was no information on the origin of the released birds in the data. To test if game bird releases affected AIV presence/absence in the passive and active AIV surveillance data, we extracted observations from 2018 to 2020 from the surveillance data. We included the year 2020, as we allowed for game bird release to have occurred up to 8 months prior to an observation in the surveillance data. For each observation in the passive AIV surveillance data, we then calculated the nearest game bird release within the last 8 months prior to the observation and identified the species released and the number of birds released. For the active and wild bird AIV surveillance data, we calculated the number of game bird releases and the total number of birds released up to 8 months prior to the observation within the same postal code as the observation.

### 2.3 Landscape variables

As wild birds are natural reservoirs of AIV and the dispersal of AIV is thus linked to wild bird movement, we wanted to include landscape variables that could be associated with wild birds, such as breeding sites, feeding sites and overwintering sites. In particular, migrating birds have long been suspected to introduce AIV into naïve populations (Hill et al., 2012; Verhagen et al., 2014), thus we focused on landscape variables (coastal areas and wetlands) where migratory birds are known to gather in high numbers (Belkhiria et al., 2018).

We obtained Corine land cover data as a 100 m^2^ resolution raster consisting of 100 × 100 m pixels (European Environment Agency, 2018). For each observation in the passive AIV surveillance data, we extracted the land cover types for the UTM coordinates using the raster package (Hijmans, 2019) in R 3.5.2 (R Development Core Team, 2018). We furthermore calculated distance to coast and distance to wetlands for the passive surveillance data in ArcMap 10.6.1 (Environmental Systems Research Institute, 2017). Distance to wetlands was calculated by selecting only Corine land cover types defined as wetlands (inland marshes, peat bogs, salt marches, salines, intertidal flats). We then calculated the closest distance from our observations to a wetland pixel centroid. To calculate distance to coast line, we obtained a shape file of the Danish coast line (Danish Map Supply; Kortforsyningen, 2020) and added a 1 km buffer. We then calculated the closest distance from our observations to this buffered coastline.

To assess the effect of accessibility on passive AIV surveillance locations, we furthermore calculated distance to roads and distance to cities as well as population density at each location. To calculate distance to roads, we obtained a shape file of all roads in Denmark (Danish Map Supply; Kortforsyningen, 2020) and calculated the closest distance to a road for each location. Population density at a location was extracted from the Gridded Population of the World dataset (raster with 1 km^2^ resolution; Socioeconomic Data & Applications Center, NASA, 2015). We also used this raster data to calculate distance to nearest city, defining a city to be a raster grid cell with ≥ 200 inhabitants/km^2^. Distance to nearest city pixel centroid was then calculated for each location in the passive AIV surveillance data. All distance calculations were completed in ArcMap 10.6.1 (Environmental Systems Research Institute, 2017).

As the active and wild bird AIV surveillance data were at the postal code level, instead of distances, we calculated the area of wetlands, coast and city within a postal code. We chose the area of city as a measure of whether the area within a postal code was mostly rural with a low density of people or if it was more densely inhabited. Area of wetland and coast were calculated using the 100 m^2^ resolution Corine land cover data (European Environment Agency, 2018), whereas area of city was calculated using the Gridded Population of the World dataset (raster with 1 km^2^ resolution; Socioeconomic Data & Applications Center, NASA, 2015). Similar to the passive AIV surveillance data calculations, a city was defined as having > 200 inhabitants/km^2^. These calculations were completed in R 3.5.2 (R Development Core Team, 2018), using the raster package (Hijmans, 2019).

### 2.4 Bias in the passive AIV surveillance data

To assess any potential bias in data submitted by the public, we compared our passive AIV surveillance data locations to random locations within Denmark in regard to accessibility. We created random locations and extracted distance to coast, distance to roads, distance to cities, and population density for each of these locations, using the same methods as in section 2.3.

### 2.5 Statistical analysis

To test for bias in the passive AIV surveillance data, we compared accessibility variables from these locations to the random generated locations using a Kolmogorov–Smirnov test in R 3.5.2 (R Development Core Team, 2018).

We used mixed generalised linear models (GLMs) in the lme4 package (Bates et al., 2015) in R 3.5.2 (R Development Core Team, 2018) to test for associations between landscape and game bird variables and passive, active and wild bird AIV surveillance data. For the passive AIV surveillance data, we used year and month of the observations as random effects, since we knew that observations varied over the months and years. We could not estimate prevalence due to the nature of the data, and our focus was on whether AIV was present at a location or not. Thus, if multiple birds from the same location were observed on the exact same date (meaning they were probably found together), we aggregated these multiple observations into a single observation with presence of AIV if any of the observations were AIV positive (see section 3.1 regarding the differentiation of subtypes in the data). Exact locations very rarely reoccurred on separate dates (see Section 3.1), and thus location was excluded as a random variable. For the active and wild bird AIV surveillance data, we also used year and month as random variables. These data were based on postal codes and the same postal codes did reoccur between months and years, thus postal code was also used as a random variable. In the active AIV surveillance data, an observation could be anything from a single bird, to a pooled sample of multiple birds. To avoid any errors or misrepresentations arising from this—and as we were only interested in whether AIV had been confirmed within a postal code in a given month—we summarized observations from the same month and postal code into one observation. If any of the multiple observations within the same month and postal code were AIV positive, the summarized observation was classified as positive (see Section 3.1). This procedure was also used on the wild bird AIV surveillance data.

Effect of game bird release was analysed for the years 2018−2020, and we included the year and month of the observations as random variables. As above, we aggregated multiple observations from the same location or postal code on the exact same date (passive AIV surveillance) or from the same month and year (active and wild bird AIV surveillance) into one single presence/absence observation. For the active and wild bird AIV surveillance data, we then calculated the number of releases and the total number of birds released up to 8 months prior to the summarized data for that month and postal code. For active and wild bird AIV surveillance data, we also included postal code as a random effect. We only used the GLM with variables pertaining to game birds, as we wanted to investigate any possible association.

When needed, for all GLMs, we used backwards stepwise elimination by removing the variable with highest P‐value, and re‐running the mixed GLM. We also performed an ANOVA between the original and the reduced model to check whether reduction in the residual sum of squares (SS) was statistically significant or not, and compared AIC‐values between models. Lastly, we checked the final models for spatial autocorrelation by plotting the residuals.

If the landscape variables were found to be associated with AIV presence, we wanted to use these variables and the GLM models to predict the probability of AIV presence throughout Denmark. To measure predictive power of our GLM models, we reran the models using a leave‐one‐out cross validation (LOOCV) scheme. This method fits the model as many times as there are observations and each time, withholds one location. We then used the model to predict the withheld location. By withholding all locations, one‐by‐one, we achieved a measure of predictive power—that is, how well we could predict the AIV status of each location based on the other locations. As the models could not predict using unknown factor levels in the LOOCV (for example unique postal code or unique Corine land cover), we had to exclude observations whose factor level only appeared once in the dataset. We did this because when leaving out an observation with a unique factor level in the LOOCV, the model based on the remaining factor levels does not recognize the one left out, and thus cannot predict using this factor level. We also investigated the predictive power by estimating accuracy, sensitivity and specificity to assess the validity of using the model to predict unknown locations.

For the passive AIV surveillance models, we wanted to predict a map of Denmark in a 1 km^2^ resolution. To do so, we created three 1 km^2^ raster maps that each covered the entire area of Denmark. We obtained Corine land cover data in a 1 km^2^ raster resolution (European Environment Agency, 2018), and removed land cover types not observed in the location data, as we would not predict to unobserved land covers. For the other two rasters, for each raster pixel centroid within the rasters, we calculated the distance to coast or to wetlands, and thus created two rasters that for each 1 km^2^ in Denmark depicted the distance to coast and the distance to wetlands respectively. We used Corine land cover (1 km^2^, European Environment Agency, 2018) to calculate the distances to coast and wetlands. For the active and wild bird AIV surveillance data, we created data on the area of coast, wetlands and city for each postal code in Denmark (based on Corine land cover 100 m^2^ resolution raster, thus the units are in 100 m^2^). All calculations were completed in R 3.5.2 (R Development Core Team, 2018).

### 2.6 Cluster analysis

To identify potential clusters of AIV within Denmark, we used the program SatScan and the package rsatscan (Kleinman, 2015) in R 3.5.2 (R Development Core Team, 2018). For passive, active and wild bird AIV surveillance data, we performed spatial scan analyses for summarized years and for separate years with an elliptical scanning window, using the Bernoulli probability model and a maximum spatial window size of less than or equal to 50% of the total population at risk. This form of analysis identifies significant spatial clusters where there is a higher (hotspots) or lower (cold spots) number of positive cases within the scanning window than expected based on the Bernoulli probability of the entire study area. SatScan then reports the ODE, which is the ratio of observed number of positive cases within a cluster to the expected number. Interpretation of an ODE of 1 means that there is no difference from the expected number of cases. We used the Gini coefficient (Han et al., 2016) for cluster selection, as it measures the heterogeneity of the cluster collection, aiding us in which clusters to report (multiple smaller clusters versus large joint clusters). All analyses focused on presence or absence of AIV at a specific site or postal code—not the number of cases reported.

## 3 RESULTS

### 3.1 Passive and active AIV surveillance

**FIGURE 1:**
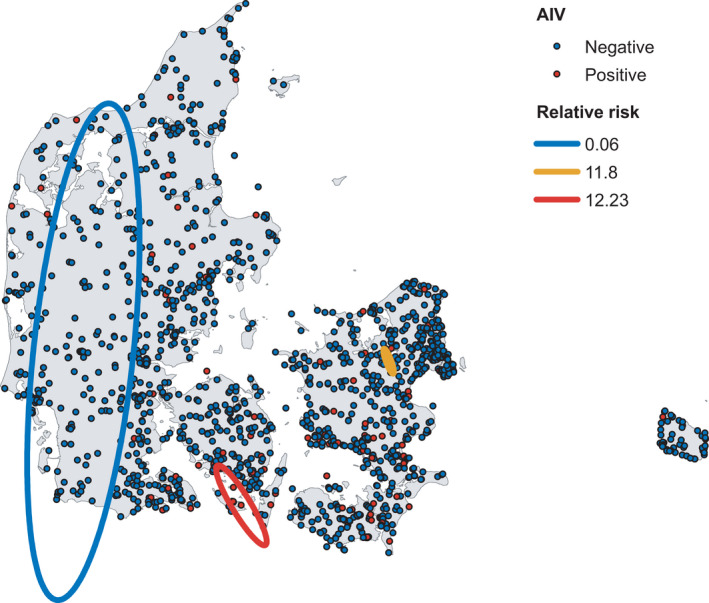
Passive AIV surveillance data and estimated clusters for the combined years 2006–2020. Clusters were analysed using SatScan on presence/absence of AIV and only significant clusters with the maximum Gini coefficient are depicted. Satscan calculates ODE, which is the observed AIV cases divided by expected AIV cases based on the Bernoulli probability of the entire study area

**FIGURE 2:**
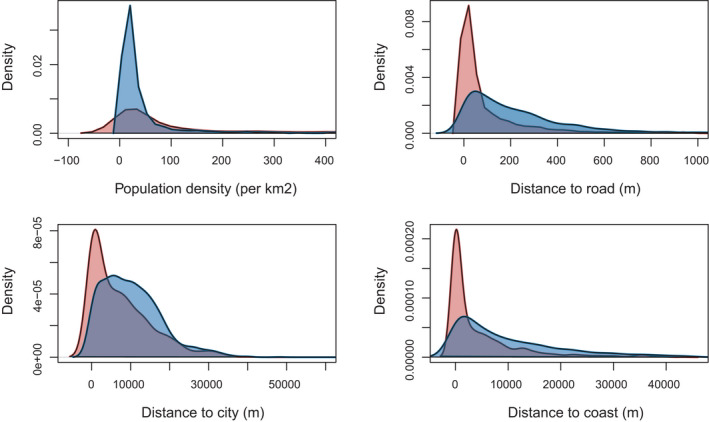
Density plots of locations recorded through passive AI surveillance in Denmark, 2006–2020 (red) and random locations in Denmark (blue) in relation to population density, distance to nearest city (≥200 inhabitants/km^2^), distance to coast and distance to nearest road. All x‐axes have been truncated to omit low density observations. As the kernel density calculations replace each observation by a small probability density, negative values around observation zeroes will occur

As only a few wild birds in the passive AIV surveillance data tested positive for AIV, we did not differentiate the positive data by AIV subtype, but rather we categorised the data as AIV detected at a location or not. The different AIV subtypes detected are summarised in Table 1. For the same reason, we did not differentiate the data by bird species. The passive AIV surveillance dataset consisted of 2,089 observation entries and 1,601 unique site locations (Figure 1). Of these 2,089 entries, 189 were AIV positive (Table 1). When summarizing same‐date and same‐location observations for the mixed GLMs, 208 of the 1,601 unique sites had multiple entries ranging from 2−55 birds. The summarized dataset used in the GLM contained 1,614 observations, as 11 locations had multiple entries on different dates within the same year (9 locations with 2 dates, and 2 locations with 3 dates, Figure S1). Of the 1,614 observations, 144 were AIV positive. We found significant differences for all accessibility variables when the 1,601 unique locations were compared to 1,601 random locations (all *p* < .0001, Figure 2), but as positive and negative AIV locations were equally biased, we proceeded with our analyses described in Section 3.3 and 3.4.

**TABLE 1:** Amount of observations in the passive and active AIV surveillance data divided into AIV subtypes. In some cases, only H5/H7 was screened for in a test positive for Influenza A virus, thus no further subtyping was performed (‘not H5/H7’)

Data	Totals	AIV subtype	# observations
Passive AIV surveillance data		H3 N2 H5 H5 N1 H5 N6 H5 N8 H7 not H5/H7	1 24 22 43 81 1 17
	Total AIV positive		189
	Total AIV negative		1900
	Total observations		2089
Active AIV surveillance data		both H5 and H7 H1 N1 H1 N2 H3 N2 H3 N8 H5 H5 N2 H6 N1 H6 N2 H7 H7 N1 H11 N9 H12 N5 not H5/H7	3 1 1 1 3 177 1 1 4 9 2 1 2 860 8,912
	Total AIV positive		1,066
	Total AIV negative		7,980
	Total observations		8,912

**FIGURE 3:**
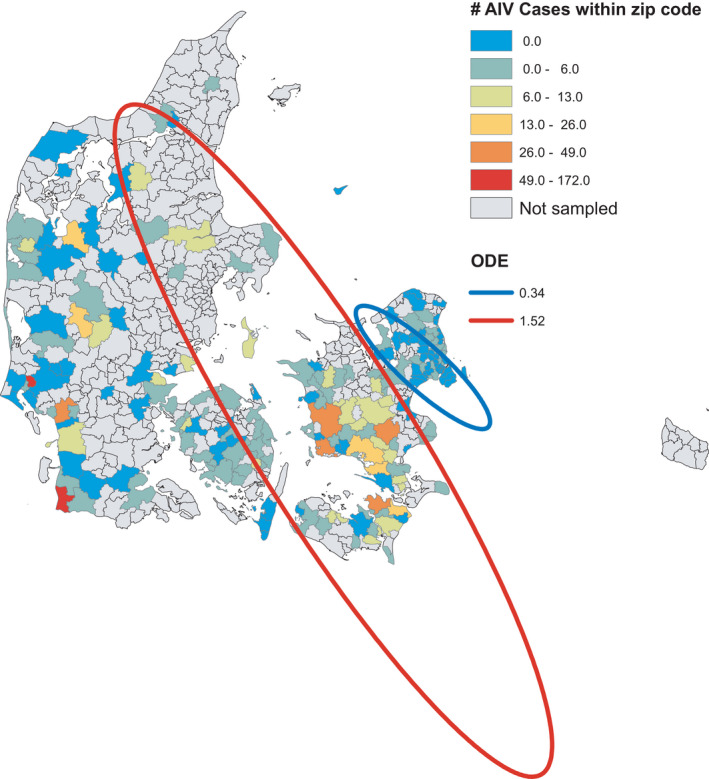
Active AIV surveillance data and estimated clusters for the combined years 2007–2019. Clusters were analysed using SatScan on presence/absence of AIV and only significant clusters with the maximum Gini coefficient are depicted. Satscan calculates ODE, which is the observed AIV cases divided by expected AIV cases based on the Bernoulli probability of the entire study area

**FIGURE 4:**
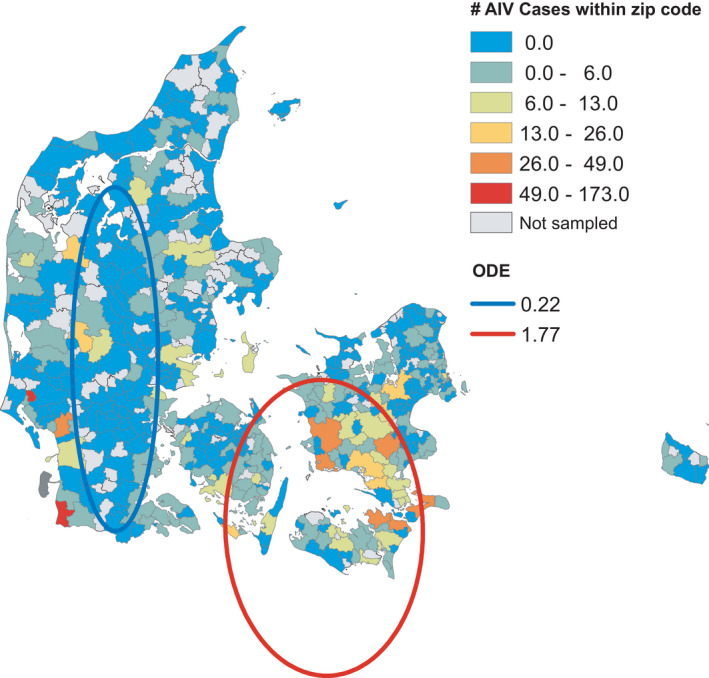
Wild bird AIV surveillance data and estimated clusters for the combined years 2006–2020. Clusters were analysed using SatScan on presence/absence of AIV and only significant clusters with the maximum Gini coefficient are depicted. Satscan calculates ODE, which is the observed AIV cases divided by expected AIV cases based on the Bernoulli probability of the entire study area

The active AIV surveillance dataset consisted of 8,912 observations within 234 unique postal codes (Figure 3). There were 1,066 observations in this dataset that tested positive for AIV (the AIV subtypes are summarised in Table 1). Summarizing over month, year and postal codes for the GLMs produced 873 observations, of which 319 were AIV positive (Figure S1). Combining all wild bird AIV surveillance data resulted in 11,001 observations within 480 unique postal codes, and 1,255 AIV positive observations (Figure 4). Summarizing this dataset over month, year and postal code produced 1,977 observations, of which 426 were AIV positive (Figure S1).

The number of observations in both the passive and active AIV surveillance data differed over the years (Figure S2) and over the months (Figure S3). For the passive surveillance, most observations were from January to April with a small peak in November and most of the positive observations were in March and November. In the active AIV surveillance data, most observations were from September to December, which were also the months with the most positive observations. Several different bird species in the surveillance data tested positive for AIV, most often duck species, swans and raptors (Figure S4).

### 3.2 Data on game birds

A total of 2,268 game bird releases were recorded from 2018−2019 at 1,179 unique locations. The total number of birds released was 1,558,302; of these 92.7% were pheasants (*Phasianus colchicus*), 6.6% were mallards (*Anas platyrhynchos*) and 0.7% were grey partridges (*Perdix perdix*).

### 3.3 Landscape and AIV presence/absence

For the passive AIV surveillance data, distance to coast and distance to wetlands were significant (*p* < .01, odds ratio (OR) = 0.9994 and 0.9992, respectively), whereas land cover at the location was not. However, we kept the land cover variable in the model, since a comparison of the full and reduced model showed significant differences in the residual SS (*p* < .0001) and removing land cover increased the AIC and reduced the R^2^ (Table 2, Figure S1). The OR indicates that for every meter increase in the distance from the coast, the likelihood of AIV presence decreases by 0.06%. This decrease in likelihood was 0.08% for wetlands (Table 2). Accounting for both fixed and random variables, the R^2^ for the full model was 0.86. For the active surveillance data, only city was significant (*p* < .01, OR = 0.9822, Table 2); the OR indicated that for every increase in the area of city (in units of 100 m^2^), the likelihood of AIV decreased by 1.78%. We selected the final model that included area of city and area of coast as variables, because this model was not significantly different from the full model (no significant differences in the residual SS, *p* <.05, same R^2^ and a reduction in AIC, Table 2, Figure S1). R^2^ was 0.52 for the final model. In the wild bird AIV surveillance data, we found that the area of coast (*p* <.01, OR = 1.0008) and the area of city (*p* <.01, OR = 0.9887) were significant. We used the reduced model without the wetlands variable, because a comparison of the full and reduced models showed no significant differences in the residual SS (*p* >.05, Table 2, Figure S1) and we observed a smaller‐AIC value and no change in the R^2^. The OR for area of coast indicates that for every unit the area of coast increases (here unit is 100 m^2^), the likelihood of AIV presence increases by 0.08%. For area of city, the OR indicates that for every increase in a unit area of city (unit is 100 m^2^), the likelihood of AIV presence decreases by 1.13%. R^2^ was 0.43 when both fixed and random variables were included. Detailed results for all mixed GLMs are shown in Table 2, and an overview of the data used and the final GLMs are shown in Figure S1. Residual plots of all final models indicated that the active AIV surveillance model had spatial autocorrelation in the residuals (Figure S5), which was further confirmed with Moran's I (*I* = 0.05, *z* = 5.70, *p* <.0001). Spatial autocorrelation of the residuals (Moran's I: *I* = 0.04, *z* = 4.71, *p* <.0001) was still present when postal code centroid coordinates were included as independent variables in the model, therefore coordinates were excluded from the final model. However, a spline (cross‐) correlogram of the final model residuals showed that the spatial autocorrelation was generally weak, with a weak negative autocorrelation (correlation coefficients <−0.20) at distances of 300 km (Figure S6). Although the spatial autocorrelation was weak, these results indicate that we did not account for all of the spatial variation within the data.

**TABLE 2:** Mixed logistic GLM results for passive, active and wild bird AIV surveillance data. The Corine land cover variable is not shown for the full passive model, as this factor variable had over 20 classes, none of which were significant. The ANOVA *p*‐values are from comparing the reduced model to the full model. The R^2^‐values depicted are Nakagawa and Schielzeth's R^2^ for mixed models from the MuMIn package (Barton, 2009) in R 3.5.2 (R Development Core Team, 2018). These values show the R^2^ for fixed variables only as well as the R^2^ for fixed and random variables combined. Abbreviations are explained in the footnote

Data	Fixed variables	*z*‐value	*p*‐value	Random variables, variance/stdev	ANOVA, *p*‐value	OR	R^2^ fixed only/all	AIC
Passive AIV	Corine LC DistToCoast, DistToWetlands	−3.31 −2.48	< 0.001 <0.05	Month: 0.0055/0.074 Year: 1.81/1.35		0.9994 0.9992	0.79/0.86	820.3
	DistToCoast, DistToWetlands	−3.98 −2.78	*p* <.0001 *p* <.01	Month: 0.03/0.18 Year: 1.85/1.36	< 0.0001	0.9999 0.9999	0.065/0.40	842.2
Active AIV	Coast Wetlands City	1.50 1.07 −2.30	0.13 0.29 *p* <.01	Month: 2.00/1.42 Year: 2.12E−10/1.42 PC: 1.79/1.34		1.0007 1.0002 0.9823	0.033/0.52	985.7
	Coast City	1.70 −2.70	0.089 *p* <.01	Month: 1.55/1.24 Year: 0.00/0.00 PC: 1.82/1.35	0.29	1.0008 0.9822	0.028/0.52	984.8
Wild birds AIV	Coast Wetlands City	2.54 0.18 −2.69	<0.05 0.86 <0.01	Month: 1.05/1.02 Year: 0.26/0.51 PC: 1.01/1.01		1.0008 1.0000 0.9887	0.020/0.43	1702.7
	Coast City	2.62 −2.69	<0.01 <0.01	Month: 1.05/1.02 Year: 0.26/0.51 PC 1.02/1.01	0.85	1.0008 0.9887	0.020/0.43	1,700.7
Game birds versus. passive AIV	Pheasant Mallard NearestRL NumBirds	−0.05 0.70 −0.35 0.28	0.96 0.48 0.73 0.78	Month: 3.37E−10/2.52E−5 Year: 0.62/0.79		0.9487 2.3342 1.0000 1.0001	0.036/0.19	86.6
	NearestRL NumBirds	−0.18 0.29	0.86 0.78	Month: 0.00/0.00 Year: 0.60/0.78	0.47	1.0000 1.0001	0.006/0.16	84.1
	NumBirds	0.35	0.73	Month: 6.99E−10/2.64E−5 Year: 0.62/0.79	0.65	1.0001	0.003/0.16	82.2
Game birds versus. active AIV	TotBirds NumRL	−1.27 1.06	0.20 0.29	Month: 1.07/1.04 Year: 0.00/0.00 PC: 0.28/0.53		0.9999 1.0641	0.026/0.31	139.9
	TotBirds	−0.77	0.44	Month: 1.10/1.05 Year: 0.00/0.00 PC: 0.21/0.46	0.32	1.0000	0.096/0.29	138.9
Game birds versus. wild bird AIV	TotBirds NumRL	−1.66 1.58	0.10 0.11	Month: 0.51/0.71 Year: 0.14/0.37 PC: 0.40/0.64		0.9999 1.0743	0.016/0.25	339.5
	TotBirds	−0.68	0.50	Month: 0.55/0.74 Year: 0.13/0.36 PC: 0.39/0.63	0.18	0.1000	0.003/0.25	339.3
	NumRL	0.10	0.92	Month: 0.53/0.73 Year: 0.13/0.36 PC: 0.38/0.62	0.13	1.0025	5.21E−5/0.24	339.8

N<del author="Lene Jung Kjær" command="Delete" timestamp="1614687738812" title="Deleted by Lene Jung Kjær on 2.3.2021 13.22.18" class="reU3">ote</del>

Abbreviations: and NumBirds, the number of birds released there. TotBirds, total amount of birds released within the postal code (up to 8 months prior to an observations) and NumRL, number of releases within that postal code; City, area of city within postal code (in units of 100 m^2^); Coast, area of coast within postal codes (in units of 100 m^2^); DistToCoast, distance to coast in meters; DistToWetlands, distance to wetlands in meters; LC, land cover; NearestRL, distance to nearest release site; OR, odds ratio; PC, postal code; stdev, standard deviation; Wetlands, area of wetland within postal code (in units of 100 m^2^).

We ran the LOOCV for the passive AIV surveillance data on 1,612 out of the 1,614 observations in the summarized dataset, as two land cover types were only found once in the dataset. The LOOCV produced an accuracy of 0.91 when using the default threshold value of 0.5 for classification (probability of AIV presence above 0.5 is classified as a presence, whereas anything below or equal to 0.5 is classified as an absence). However, this accuracy equalled the proportion of AIV negative observations in the data, meaning that the model was not better than predicting all observations to be AIV negative. Hence, the model sensitivity was 0 and the specificity was 1, meaning that none of the positive observations were classified as positive. We could change the threshold to obtain a higher sensitivity (which would then lower the specificity), but we were not able to obtain an accuracy higher than the proportion of absences (0.91). Thus, predictions for this model should be viewed with caution.

For the active AIV surveillance model, the LOOCV was performed on 801 observations, as 72 of the observations in the summarized dataset (*n* = 873) had postal codes that appeared only once. With a threshold of 0.5, the active model had an accuracy of 0.77, a sensitivity of 0.62 and a specificity of 0.85. As the proportion of absences (majority class) was 0.65, this model performed better than if all observations were predicted to be absences. We performed LOOCV on 1,836 out of the 1,977 observations in the summarized wild bird AIV surveillance dataset, as 141 postal codes only appeared once in the summarized dataset. With a default threshold value of 0.5, the accuracy was 0.82, with a sensitivity of 0.32 and a specificity of 0.96. Here the proportion of absences was 0.78, thus the model was more informative than a model only predicting absences.

**FIGURE 5:**
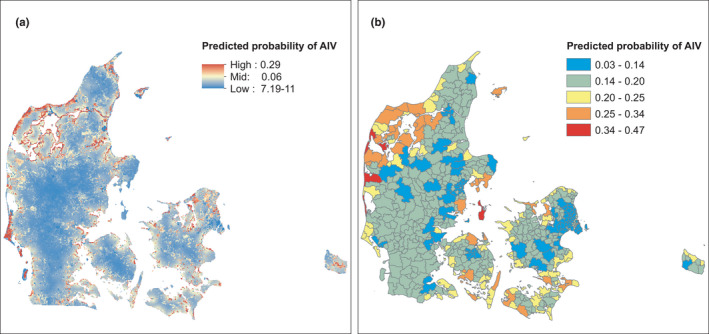
Predicted probabilities of AIV presence, based on the a) the passive AIV surveillance data model with variables land cover, distance to coast and distance to wetlands, and b) the active AIV surveillance data with variables area of coast and area of city

For the full passive AIV surveillance model, we used the Corine 1 km^2^ land cover data and the coast‐ and wetlands‐distance rasters to predict the probability of AIV throughout Denmark. We set the random effects to zero to predict over all the years and the months. We found high‐risk areas along the coast and around the fjords (Figure 5a). We also used the active AIV surveillance model to predict the probability of AIV presence based on postal code level area of coast and area of city. We selected the active AIV surveillance model—rather than the wild bird surveillance model, as the sensitivity was higher, thus predicted positive postal codes were more likely to be correctly classified in this model than in the wild bird surveillance model. Again, we set the random variables to zero to predict over all the years, months and postal codes. Here we also found the highest probabilities of AIV presence in postal codes with coastline or along fjords (Figure 5b).

For the game bird release data, we found no significant association with distance to bird release site, bird species released or number of birds released in the passive AIV surveillance data (Table 2). We also did not find any significant association with number of releases and total number of birds released in the active and wild bird AIV surveillance data (Table 2).

### 3.4 Cluster analysis

The SatScan analysis detected several significant clusters for the passive, active and wild bird AIV surveillance data. For all the AIV surveillance data, hotspots were mostly found in the southern parts of Denmark (Southern Zealand, Lolland/Falster and Funen), whereas cold spots were found in northern Zealand and Jutland (see summarised results in Figures 1, 3 and 4). For the individual years, not all years had detectable clusters or the amount of data were insufficient to perform cluster analysis (See Figures S7, S8 and S9 and Table S1). The summarized presence/absence data used for the cluster analyses are shown in Table S1.

## 4 DISCUSSION

We analysed 11,001 observations from the Danish AIV surveillance program collected from 2006 to 2020 and found associations between landscape variables and AIV presence. Furthermore, we detected spatial hot and cold spots of AIV presence within Denmark. We found differences in AIV presence across months of the year and between years. A higher number of positive samples in the active AIV surveillance were found from September to November; however, these were also the months in which most observations occurred. Many of the observations in the active AIV surveillance data originated from hunted birds, thus the higher number of observations from September to December was expected as this time period coincides with the hunting season of many Danish bird species (The Danish Hunting Association, 2020). For the passive AIV surveillance data, most positive samples were found between March and November. This coincides with the timing of bird migration, when migratory birds are in transit through Denmark (DOF BirdLife, 2020). We found the largest number of observations in 2006. This coincides with the first outbreak of HPAI in wild Danish birds (Bragstad et al., 2007), an outbreak that occurred in several European countries and caused the EU to fund compulsory passive surveillance for AIV in dead or sick wild birds and active surveillance in apparently healthy wild birds in all member countries (European Commisson, 2020; Hesterberg et al., 2009). The compulsory active surveillance in apparently healthy wild birds lasted until 2011, after which the Danish authorities continued active AIV surveillance in wild birds, albeit at a smaller scale (Hjulsager et al., 2018). The increase and decrease in the intensity of surveillance efforts can be seen in the increasing number of observations in the active AIV surveillance data from 2007−2010, and the subsequent decrease in the number of observations from 2011−2020. The most sampled species were buzzard, swans and mallards, and the distribution of sampled species showed large variation, reflecting public interest and accessibility to bird habitats. Therefore, it was not possible to quantify the risk of testing positive for AIV for the different species.

We found that the passive AIV surveillance data were biased regarding the geographical location of sample sites. The majority of recorded locations were within 35 km of a larger city and within 500 m of roads. Public access to Danish beaches might also explain numerous records close to the coast, suggesting that accessibility to wildlife areas biases the Danish passive surveillance data. However, passive surveillance is not easy to control as it depends on the willingness and efforts of the general public. Implementation of information campaigns can be of great assistance to reinforce sampling in areas with sparse information or hotspots, and would be a valuable contribution to the ongoing surveillance programme.

For the passive AIV surveillance data, we found that distance to coast and distance to wetlands were significantly associated with the presence of AIV. For the active AIV surveillance data, we furthermore found an association with the area of coast and the area of city. Other studies have found effects of landscape variables and anthropogenic factors on AI presence in both wild and domestic birds. In Thailand, Paul et al. (2010) found a positive effect of free grazing ducks, high rice‐cropping intensity areas, densely populated areas, short distances to a highway junction, and short distances to large cities on AIV presence in poultry. Gilbert et al. (2008) identified duck abundance, human population density, and rice cropping intensity as risk factors in South East Asia. In Romania, Ward et al. (2008, 2009) found associations between distance to migratory waterfowl sites, distance to major roads and distance to rivers or streams and HPAI outbreaks. Using a machine learning (ML) approach, Belkhiria et al. (2018) found spatial risk areas for AIV in wild birds in California, where land cover and distance to coast were some of the most important predictors in their model. The poor performance of our passive AIV surveillance model and the relatively low sensitivity of our active and wild bird AIV surveillance models, indicate that other factors not considered in this study might be important for predicting AIV presence. We did attempt to use ML methods on the summarised data and included environmental MODIS variables. However, this did not improve the models, and thus the simple GLMs were chosen to make prediction maps of AIV (See Data S1 for the ML description and results). Migratory birds have long been suspected of spreading AIV between regions (van der Kolk, 2019; Sullivan et al., 2018) and adding data on bird migration to our models could potentially be of value. However, no fine‐scale data are available on bird migration routes within Denmark that would enable us to distinguish between individual locations within the same region. We found no significant association between game bird releases and the passive and active surveillance. This could mean that the current legislation with testing and quarantine for imported game birds is effective to prevent transmission of AIV to wild birds. Our results could also be explained by a lack of data from other years, as we could only perform our analyses for the years 2018−2020.

Our cluster analyses identified several hot and cold spots for AIV presence within Denmark. We generally found hotspots in the southern parts of Denmark, whereas cold spots were found in northern Zealand and Jutland. The southern parts of Denmark lie on the main migration routes of duck and geese (Bregnballe et al., 2003), and the Wadden Sea along the south western coast of Denmark is also a well‐known stop‐over for migratory birds (Lotze, 2005). Thus, it is surprising that we found no hotspots in the western part of the country. This could be due to biased sampling, as only few people venture into the Wadden Sea region, and dead birds are quickly washed away. It could potentially also be due to the origin of migrating birds in the different regions of Denmark (for example from Siberia, which is known to be a hot spot for the dispersal of AIV, Lai et al., 2016; Li et al., 2014). However, as Denmark is embedded in the East Atlantic Flyway, with many different migrating birds of different origins (Bregnballe et al., 2003; Lotze, 2005), it can be difficult to determine these origins as there is no precise information on migration routes within the country.

Our predictive maps of AIV in Denmark identified high‐risk areas located around the coast and fjords in Denmark. This suggests that any potential risk‐based surveillance in wild or domestic birds should be concentrated in these areas, particularly high‐risk areas that are not extensively covered in the present Danish AIV surveillance, such as the coast and Fjords in northern Jutland. The cluster analysis found hotspots in the southern parts of Denmark, areas that our predictive maps also highlight as being high‐risk. These areas should also be included in risk‐based surveillance. Knowing which parts of Demark constitute high‐risk areas for potential AIV introduction might aid the selection of sites for new poultry facilities. Organic‐ or free‐ranging poultry farms—where the farmed birds can come into contact with wild birds—are of particular concern and any location of such farms in high‐risk areas should be avoided. It is important to note that although we did not divide any of our analyses into AIV subtypes, the majority of subtypes in the passive AIV surveillance data belonged to the HPAI types, whereas the majority of subtypes in the active AIV surveillance data belonged to the LPAI types (Table 1). Thus, our separate passive and active AIV data models can approximately be interpreted as predicting the risk of HPAI and LPAI occurrence respectively.

At the beginning of November 2020, a HPAI positive peregrine falcon (*Falco peregrinus*) was found dead near Sakskøbing on the island of Lolland in the southern parts of Denmark, an observation not included in our datasets (Ministry of Environment & Food of Denmark, 2020). This finding marked the beginning of a series of HPAI virus detections in wild birds in Denmark that are still continuing in 2021. The area where the falcon was found coincides with high‐risk areas predicted by both our passive and active AIV surveillance models. Furthermore, mid‐November 2020, there was an outbreak of HPAI in a poultry farm east of Randers in Jutland (Ministry of Environment & Food of Denmark, 2020), also an observation not included in our datasets. This particular poultry farm kept all their animals indoors, with little risk of contact to wild birds. The area, where the poultry farm was situated, coincides with predicted low risk areas in both the passive and active surveillance model, but it is important to emphasize that prediction maps based on wild bird surveillance are not expected to accurately predict the AIV risk in poultry farms, especially farms with no direct wild bird contact.

The results of our study highlight some of the deficiencies in the current Danish AIV surveillance program. The aim of the active AIV surveillance programme is mainly to study LPAI virus epidemiology whereas the aim of passive AIV surveillance programme is the early detection of HPAI viruses. Despite the different objectives of the programs, more knowledge on the epidemiology and transmission of both LPAI and HPAI demands thorough coverage of Denmark in order to be able to determine variables important for transmission and dispersal. Both our passive and active AIV surveillance models predicted high probabilities of AIV occurrence in the north‐western parts of Denmark; an area that is one of the least covered areas in the active surveillance program. More knowledge on AIV presence in these areas is needed, and our findings may elicit implementation of more thorough surveillance in these north‐western parts of Denmark. A new Animal Health Law (Regulation (EU) 2016/429, European Union, 2019) takes effect on 21 April, 2021 within the EU. Some of the main changes to the Danish AIV surveillance are that LPAI is no longer a notifiable disease, while surveillance is still mandatory. Furthermore, a risk based approach can be used for surveillance of HPAI in species not showing obvious clinical signs (duck, geese and gamebirds of the order Anseriformes), or for LPAI in poultry. For HPAI, the focus is on specified high‐risk zones, while for LPAI the focus is on areas with poultry farms, where clusters of LPAI have previously been found. Prediction maps, as presented in this paper for Denmark, can be used to pinpoint areas with elevated risk of LPAI and HPAI and thus aid in adjusting the surveillance program to adhere to the new Animal Health Law, potentially by dividing regions into high‐risk and low‐risk HPAI zones. However, the sparse data on AIV occurrence in Denmark and the variation in surveillance over the years, makes generalising over our results difficult. Moreover we were not able to conduct analyses of individual subtypes of AIV. More comprehensive studies and analysis demand more consistent sampling and a stratified sampling scheme for the future surveillance of AIV.

## 5 ETHICS STATEMENT

The authors confirm that the ethical policies of the journal, as noted on the journal's author guidelines page, have been adhered to. No ethical approval was required as this article does not use original research data, but data obtained through the Danish authorities.

## CONFLICT OF INTEREST

The authors declare no conflict of interest.

## Supporting information

Supplementary Material

## Data Availability

Data is subject to confidentiality and is not freely available.
