# Supplementary material for: Landscape effects and spatial patterns of avian influenza virus in Danish wild birds, 2006–2020
Source: Transbound Emerg Dis. 2021 May 6;69(2):706–19. doi: 10.1111/tbed.14040 (PMC9291307; doi:10.1111/tbed.14040)
Supplement: Supplementary file 1 — Supplementary Material [file TBED-69-706-s001.docx]

**Landscape effects and spatial patterns of avian influenza virus in Danish wild birds, 2006-2020**

**Supplementary file**

Lene Jung Kjær^1*^, Charlotte Kristiane Hjulsager ^2^, Lars Erik Larsen^1^, Anette Ella Boklund^1^, Tariq Halasa^1^, Michael P Ward^3^, and Carsten Thure Kirkeby^1^

^1^Section for Animal Welfare and Disease Control, Department of Veterinary and Animal Sciences, Faculty of Health and Medical Sciences, University of Copenhagen, Frederiksberg, Denmark

^2^Department of Virus & Microbiological Special Diagnostics, SSI, Copenhagen, Denmark

^3^ Faculty of Science, Sydney School of Veterinary Science, The University of Sydney, Camden NSW, Australia

**Table S1.** Summarised AIV presence/absence data for the passive, active and wild bird AIV surveillance data used in the SatScan cluster analysis.

| **AIV surveillance data** | **Year** | **# of presence/absence observations** |
| --- | --- | --- |
| Passive | All years combined | 143/1458 |
|  | 2006 | 37/739 |
|  | 2007 | 0/137 |
|  | 2008 | 0/59 |
|  | 2009 | 1/36 |
|  | 2010 | 3/36 |
|  | 2011 | 1/22 |
|  | 2012 | 1/16 |
|  | 2013 | 0/8 |
|  | 2014 | 0/9 |
|  | 2015 | 1/21 |
|  | 2016 | 44/161 |
|  | 2017 | 17/135 |
|  | 2018 | 35/127 |
|  | 2019 | 3/90 |
|  | 2020 | 0/5 |
| Active | All years combined | 103/130 |
|  | 2007 | 2/7 |
|  | 2008 | 18/50 |
|  | 2009 | 39/94 |
|  | 2010 | 28/71 |
|  | 2011 | 22/58 |
|  | 2012 | 22/48 |
|  | 2013 | 21/60 |
|  | 2014 | 17/56 |
|  | 2015 | 22/48 |
|  | 2016 | 14/54 |
|  | 2017 | 25/49 |
|  | 2018 | 20/34 |
|  | 2019 | 19/41 |
| Wild birds | All years combined | 185/462 |
|  | 2006 | 19/299 |
|  | 2007 | 2/88 |
|  | 2008 | 18/85 |
|  | 2009 | 39/109 |
|  | 2010 | 30/75 |
|  | 2011 | 23/72 |
|  | 2012 | 23/60 |
|  | 2013 | 21/60 |
|  | 2014 | 17/57 |
|  | 2015 | 23/64 |
|  | 2016 | 44/136 |
|  | 2017 | 39/145 |
|  | 2018 | 46/122 |
|  | 2019 | 22/106 |
|  | 2020 | 0/5 |

**Figure S1.** Overview of the total amount of data, the data used to run GLMs and the final GLM models for the passive, active and wild bird AIV surveillance data. PC = postal code, Corine LC = Corine land cover, DistToCoast = distance to coast in meters, DistToWetlands = distance to wetlands in meters, Coast = area of coast within postal codes (in units of 100 m^2^), City = area of city within postal code (in units of 100 m^2^).


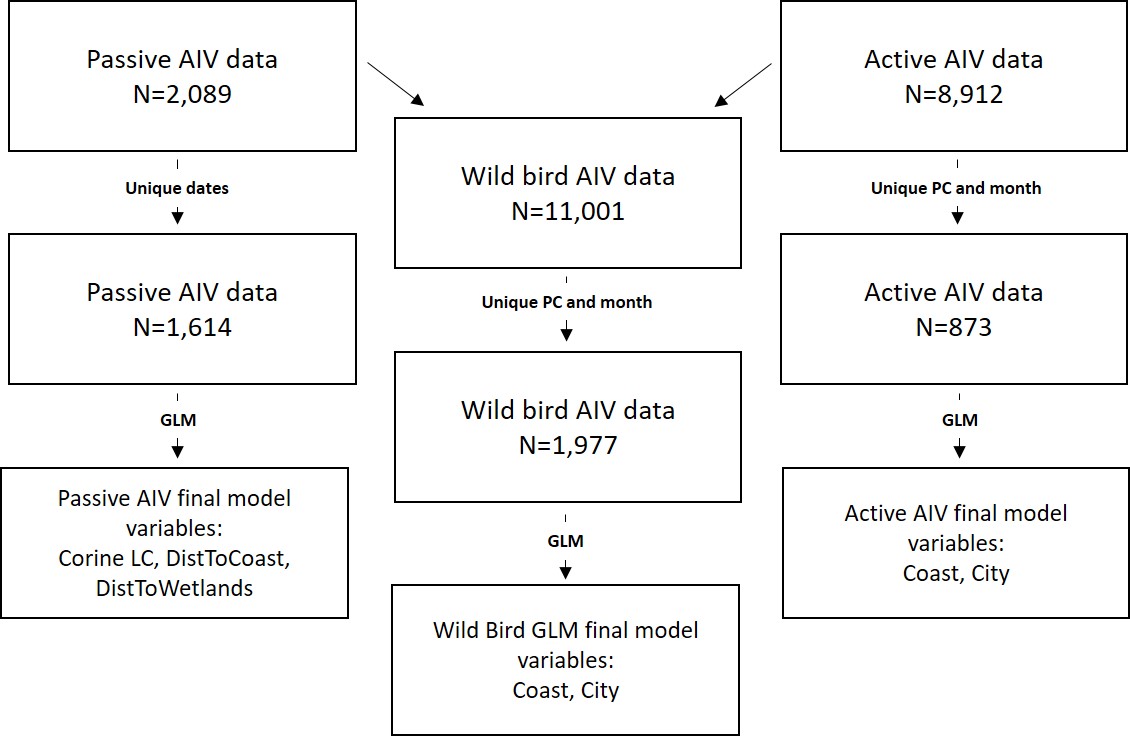


**Figure S2.** Yearly number of observations and AIV diagnosis results from the Danish A) passive AIV surveillance program (2006-2020), B) active AIV surveillance program (2007-2019).


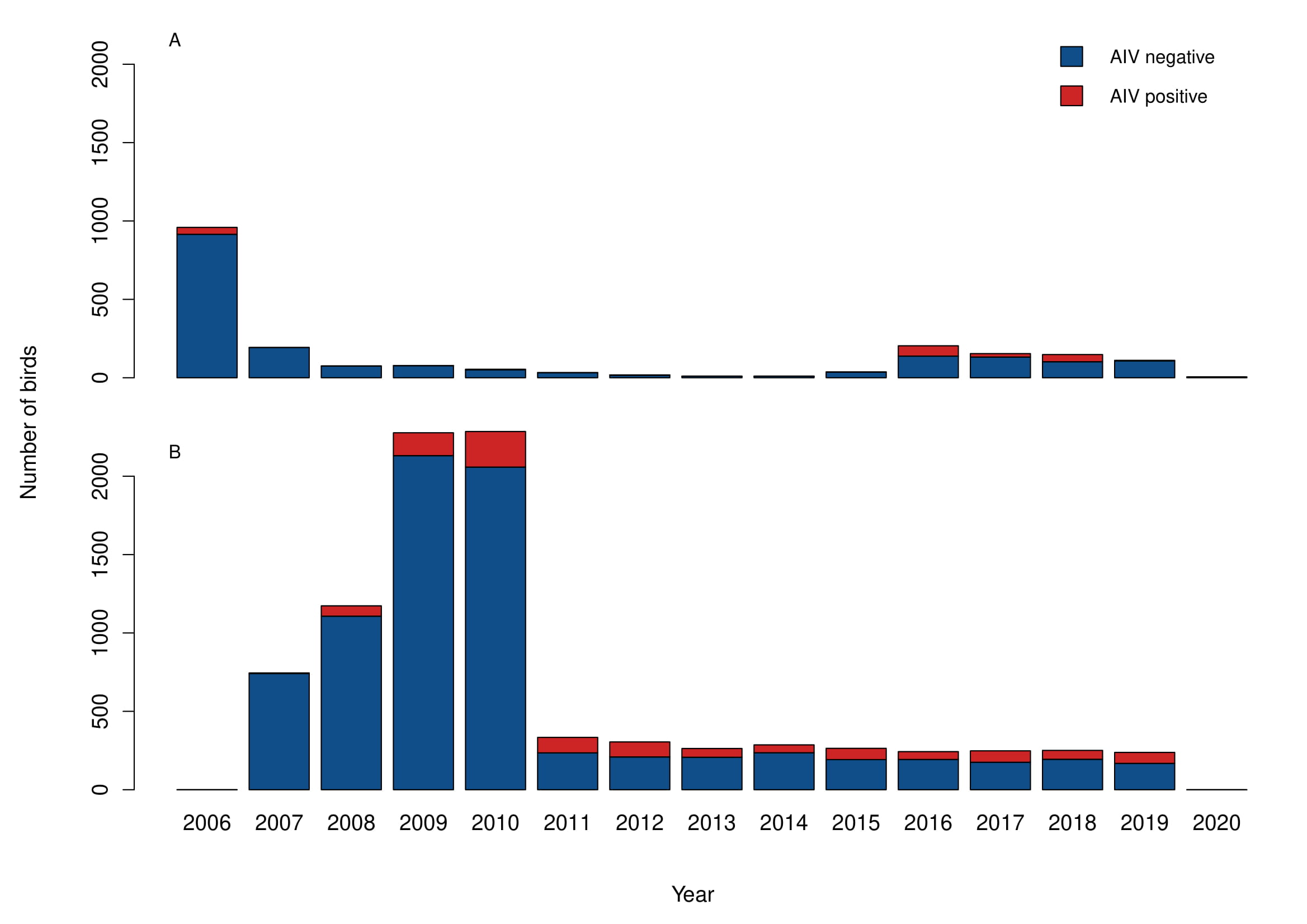


**Figure S3.** Monthly number of observations and AIV diagnosis results from the Danish A) passive AIV surveillance program (2006-2020), B) active AIV surveillance program (2007-2019).


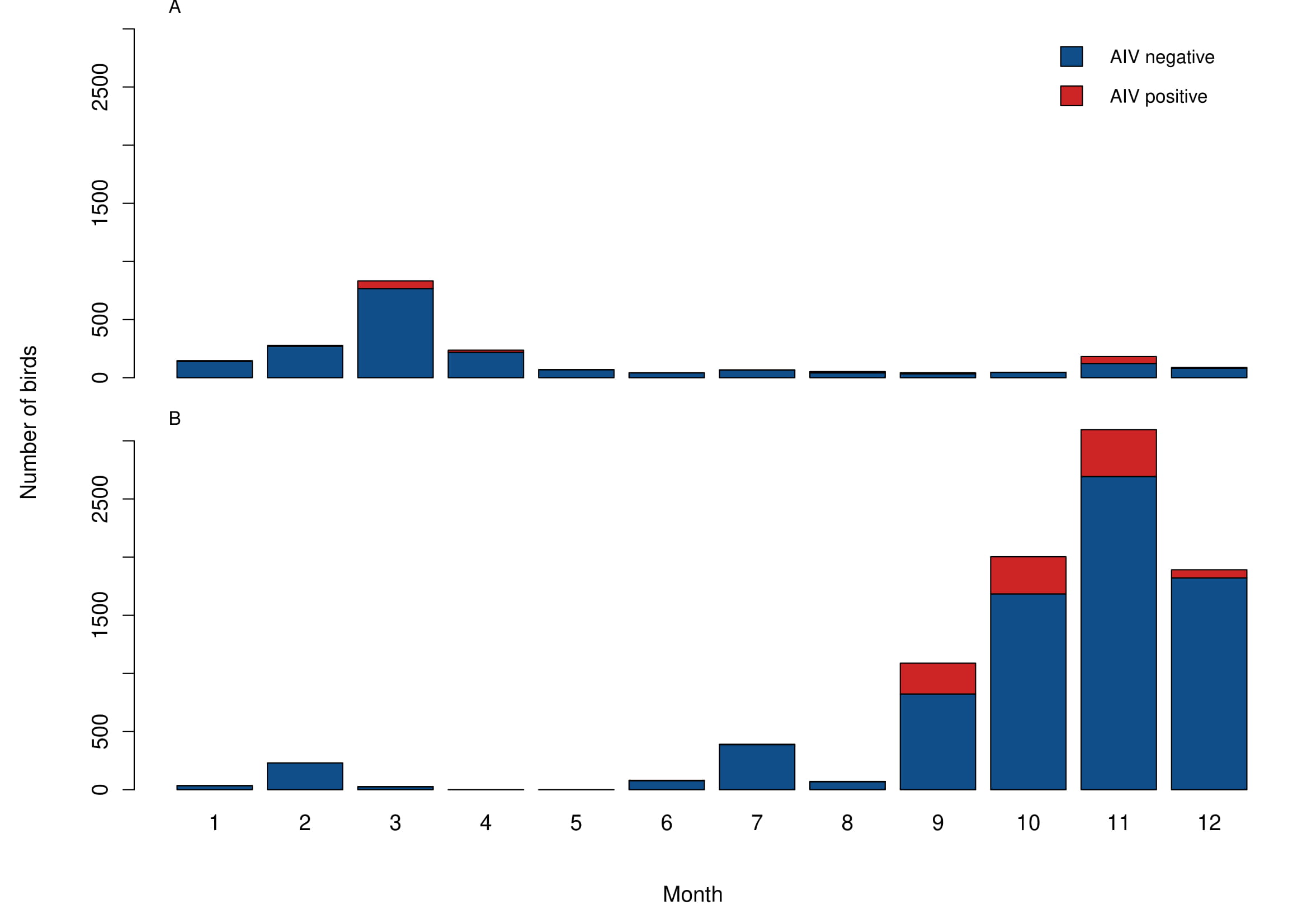


**Figure S4.** Recorded bird species and AIV diagnosis results from the Danish A) passive AIV surveillance program (2006-2020), B) active AIV surveillance program (2007-2019). Only species with at least one positive AIV diagnosis are depicted.

**
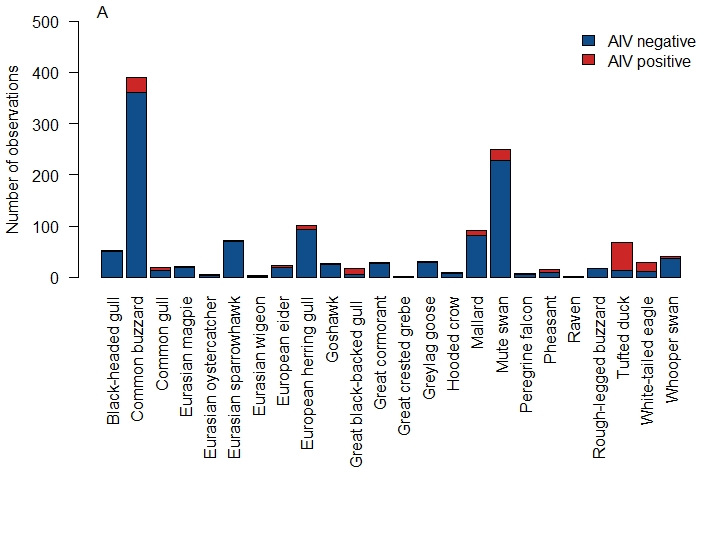
**

**
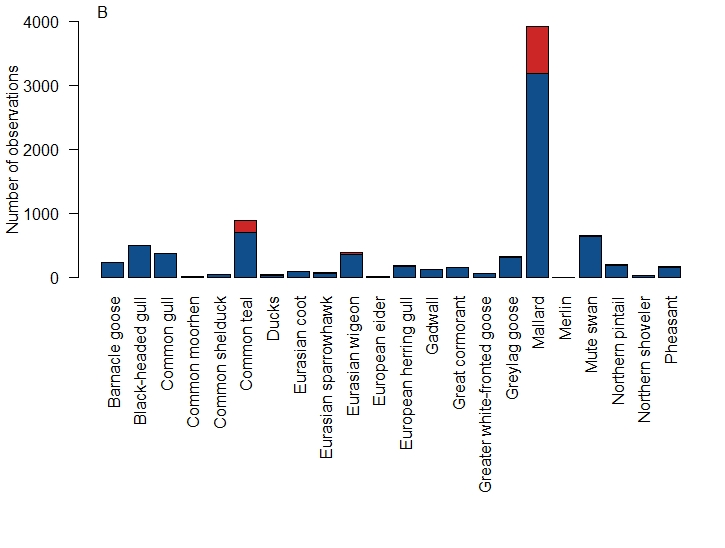
**

**Figure S5.** Residual plots of A) the passive AIV surveillance data, B) the active AIV surveillance data, and C) the wild bird AIV surveillance data, based on the final mixed GLM’s to predict AIV occurrence in Denmark.

**
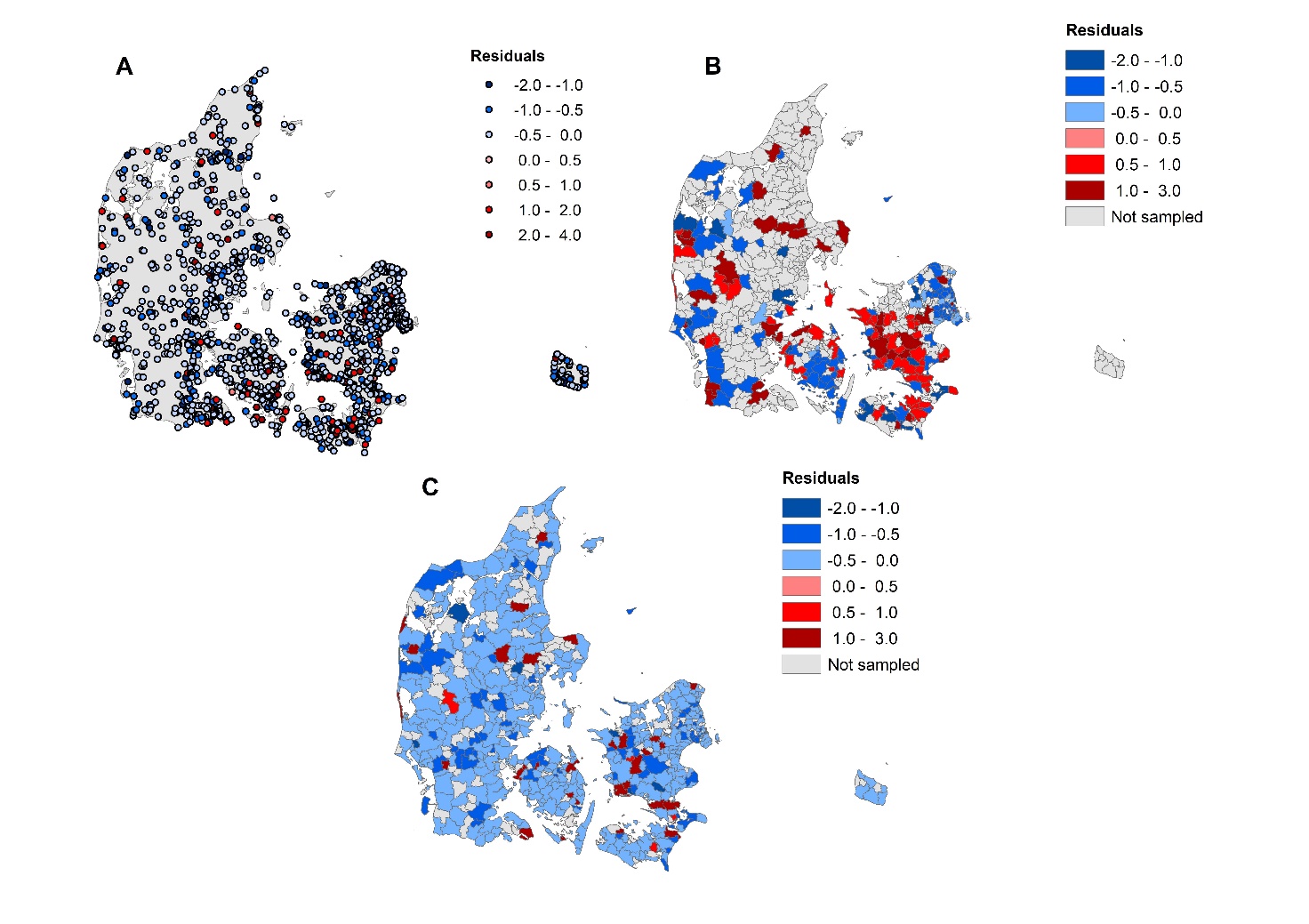
**

**Figure S6.** Spline (cross-) correlogram of the active AIV survelliance model residuals showing the spatial autocorrelation between residuals and their bootstrapped 95% confidence intervals at distances up to 350 kilometers. The Spline correlogram was calculated using centroid coordinates of each postal code region and the ncf package (Bjornstad, 2020) in R 3.5.2 (R Development Core Team, 2018).


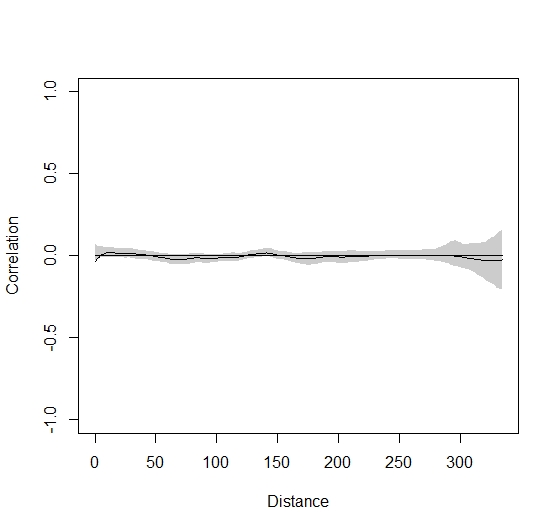


**Figure S7.** Yearly observations of AIV (LPAI and HPAI) and estimated clusters for the passive AIV surveillance data, 2006-2020. Clusters were analysed using SatScan on presence/absence of AIV and only significant clusters with the maximum Gini coefficient are depicted. Satscan calculates ODE, which is the observed AIV cases divided by expected AIV cases based on the Bernoulli probability of the entire study area.


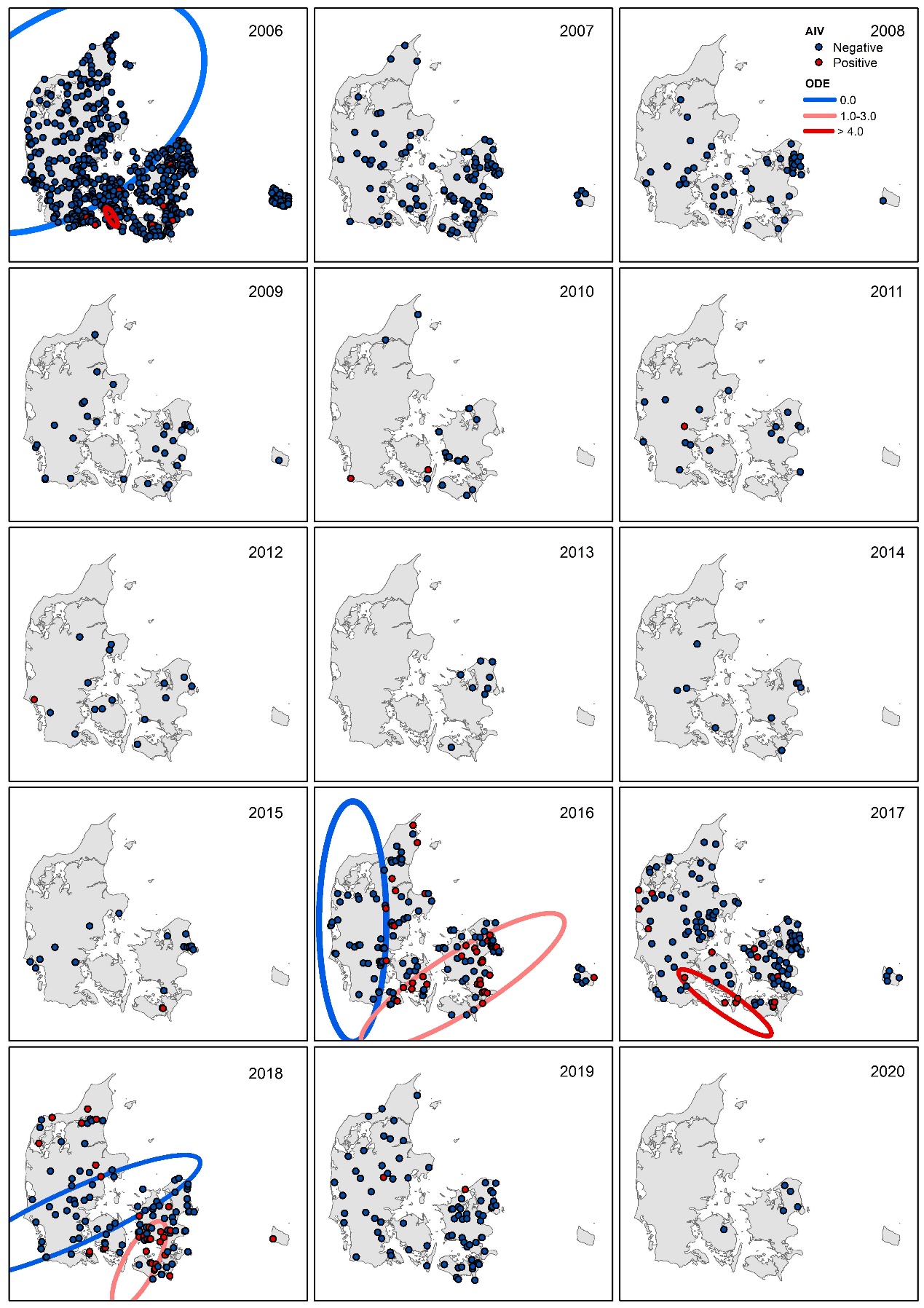


**Figure S8.** Yearly observations of AIV (LPAI and HPAI) and estimated clusters for the active AIV surveillance data, 2007-2019. Clusters were analysed using SatScan on presence/absence of AIV and only significant clusters with the maximum Gini coefficient are depicted. Satscan calculates ODE, which is the observed AIV cases divided by expected AIV cases based on the Bernoulli probability of the entire study area.


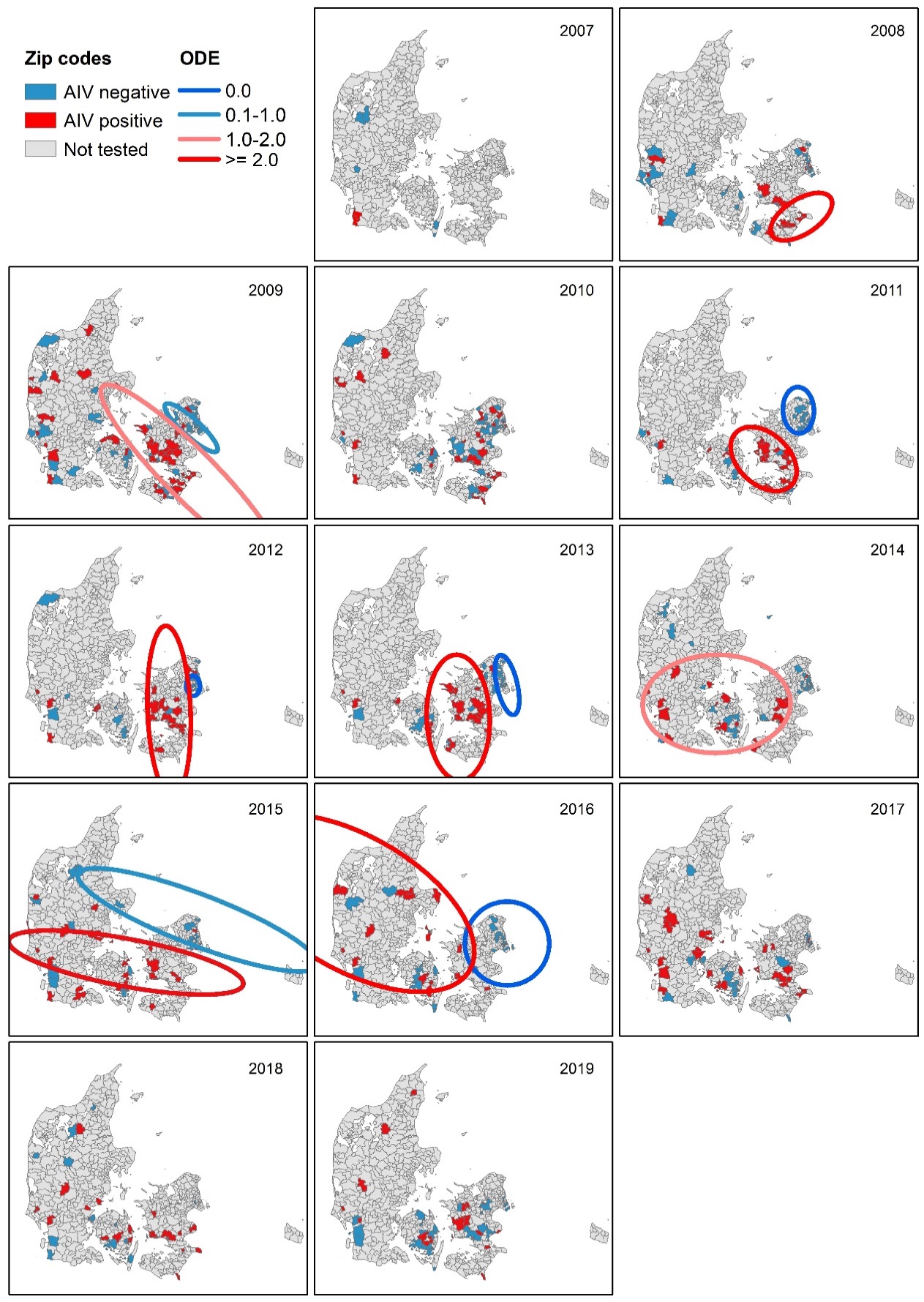
**Figure S9.** Yearly observations of AIV (LPAI and HPAI) and estimated clusters for the wild bird AIV surveillance data, 2006-2020. Clusters were analysed using SatScan on presence/absence of AIV and only significant clusters with the maximum Gini coefficient are depicted. Satscan calculates ODE, which is the observed AIV cases divided by expected AIV cases based on the Bernoulli probability of the entire study area.


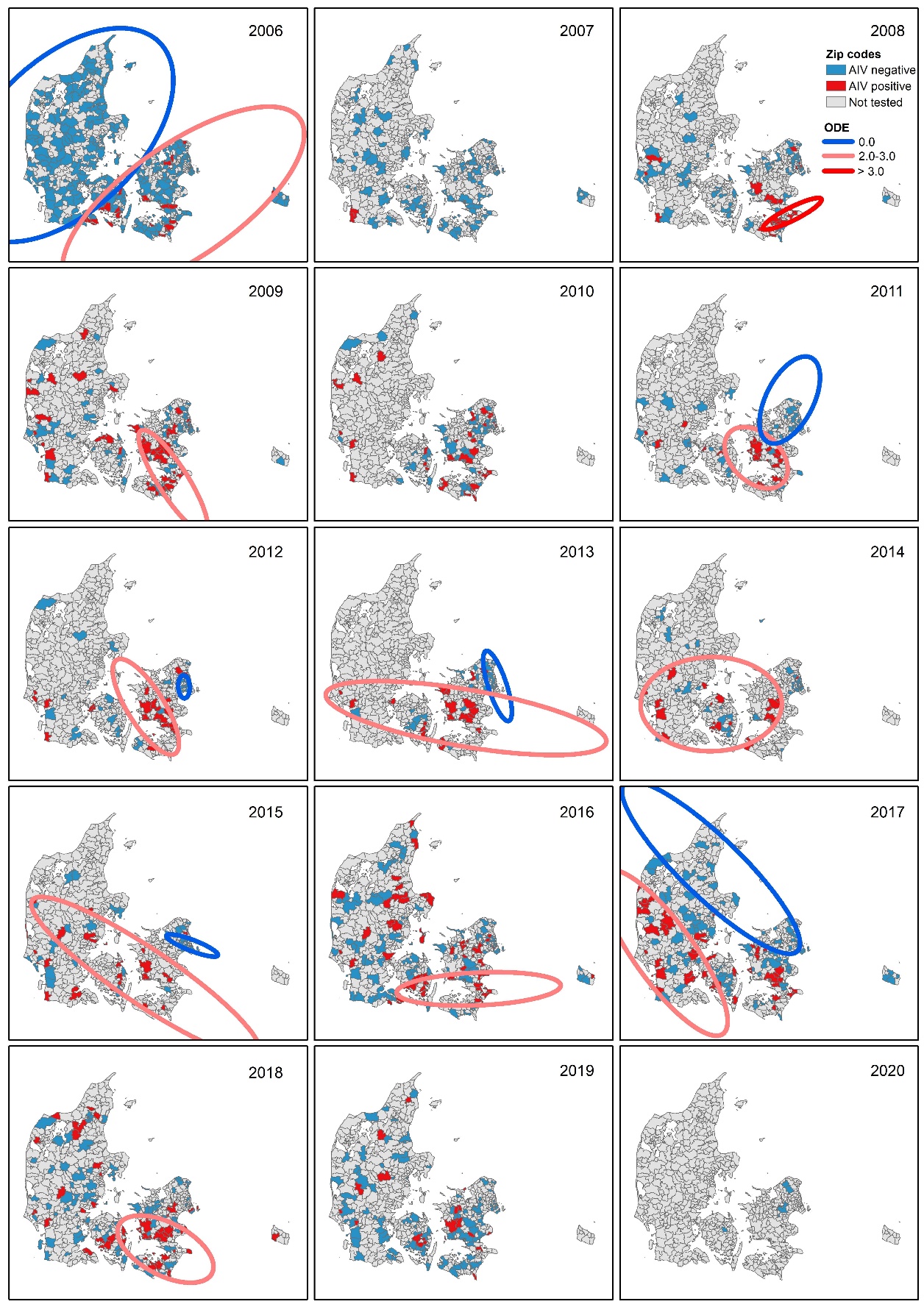


**Machine learning approach to model and predict AIV within Denmark**

We ran boosted regression trees (BRT) in R 3.5.2 (R Development Core Team, 2018) using the packages caret (Kuhn. et al., 2018) and gbm (Greenwell et al., 2019). For all our AIV surveillance data, we used the summarised data, where all locations and postal codes were unique and were designated presence/absence of AIV if AIV was found at that location/postal code at any point in time (All years combined in Table S1). We did this as BRT do not incorporate random effects, and thus we could not use multiple observations from the same location for different months and years. Therefore, the data used to construct these models are different than for the simple GLM’s described in the main manuscript.

For the passive AIV surveillance data, we used the landscape predictors described in the manuscript: distance to wetlands, distance to coast, and Corine land cover (European Environment Agency, 2018), whereas we used area of coast, area of wetlands and area of cities for the active and wild bird AIV surveillance data, In addition, for the passive AIV surveillance data, we included Fourier transformed environmental data derived from images from a MODIS satellite v5 time series for 2001-2012. We only included environmental MODIS predictors for the passive AIV surveillance data, as these predictors were not available on a postal code level, but only in a resolution of 1km^2^.

The predictors obtained from MODIS were middle infra-red index, daytime land surface temperature, night time land surface temperature, the normalised difference vegetation index, and the enhanced vegetation index. We used Fourier processed outputs for each of the variables: mean, minimum, maximum, variance in raw data, combined variance in annual, bi-annual, and tri-annual cycles as well as amplitude, phase and variance of annual bi-annual and tri-annual cycle (EDENext, 2014). We furthermore used average monthly data on altitude, temperature and precipitation from Worldclim and Bioclim (WorldClim, 2005). For details about the used environmental predictors see Kjær et al. 2019.

We assumed our data to be Bernoulli distributed (logistic regression for 0-1 outcome), and ran a 5-fold repeated cross validation (CV) scheme with 10 repetitions to estimate the prediction error and to validate our models. This methods divides our data into 5 subsets, where one subset is withheld at each run of the model, and the model is then used to predict the “unknown” data in the withheld subset. We also assessed the area under the curve (AUC) for the receiver operating characteristic (ROC) (Elith et al., 2008), and used a tuning grid to optimise model parameters, interaction depth, learning rate and minimum observations per node (Elith et al., 2008). We kept the number of trees constant at 1,500 trees. As the performance of a BRT model may be influenced by class imbalance (Batista et al., 2004), we compared models for unbalanced data to models using different balancing methods; down-scaling, up-scaling, rose (package ROSE, Lunardon et al., 2014), smote (Batista et al., 2004), tomek with the majority removed and tomek with both classes removed (Batista et al., 2004). We compared the AUC scores for each balancing method (as well as an unbalanced model), using the 5-fold repeated CV scheme for each method and chose the model with the highest AUC score to be the final model.

In our passive AIV surveillance data, 92.4% of the data were AIV negative whereas 0.076% of the data were AIV positive. The model with the highest AUC-score was the model using the SMOTE balancing method (Table S2). This model produced an accuracy of 0.85, a sensitivity of 0.34 and a specificity of 0.90 (Table S2). However, the accuracy of this passive AIV surveillance model, was lower than the proportion of the majority class (AIV negative), thus the model performed worse than a model where all predicted observations were predicted to be AIV negative (all other balancing methods produced similar results regarding accuracy). In our active AIV surveillance data, 44.20% of the data were AIV negative whereas 55.8% of the data were AIV positive, thus, no balancing method was needed. The final model had an AUC of 0.63, an accuracy of 0.65, a sensitivity of 0.76 and a specificity of 0.51 (Table S2). 61.5% of the wild bird AIV surveillance data were AIV negative with 38.5% of the data being AIV positive, and here we also did not use a balancing method. The final model produced an AUC of 0.60, an accuracy of 0.64, a sensitivity of 0.29 and a specificity of 0.85 (Table S2).

None of these models performed considerably better than the simple mixed GLM’s described in the main manuscript, thus we used the simple models to predict AIV occurrence within Denmark.

**Table S2.** AUC scores for BRT models with different balancing methods for the passive, active and wild bird AIV surveillance data. Tomek-both is the tomek method with both classes removed to balance the data, whereas tomek-majority is the tomek method where only data from the majority class are removed to obtain balancing of the data. Model results are from the 5-fold CV of the best (or only) model. Only results from the final balancing model is shown for the passive AIV surveillance model.

| **Data** | **Balancing method** | **AUC** | **Model results** | **Parameters of final model** | |
| --- | --- | --- | --- | --- | --- |
| Passive AIV surveillance | Unbalanced  Down-scaled  ROSE  SMOTE  Tomek-both  Tomek-majority  Up-scaled | 0.70  0.69  0.51  0.71  0.67  0.70  0.70 | Accuracy:0.85  Sensitivity: 0.34  Specificity: 0.90 | Interaction depth: 1  Learning rate: 0.01  Min. observations in node: 3 | |
| Active AIV surveillance | Unbalanced | 0.63 | Accuracy: 0.65  Sensitivity: 0.76  Specificity: 0.51 | Interaction depth: 1  Learning rate: 0.01  Min. observations in node: 2 |  |
| Wild bird AIV surveillance | Unbalanced | 0.60 | Accuracy: 0.64  Sensitivity: 0.29  Specificity: 0.85 | Interaction depth: 1  Learning rate: 0.01  Min. observations in node: 2 |  |

**References**

Batista, G.E.A.P.A., R.C. Prati, and M.C. Monard, 2004: A Study of the Behavior of Several Methods for Balancing Machine Learning Training Data. *ACM SIGKDD Explor. Newsl.* **6**, 20–29, DOI: 10.1145/1007730.1007735.

Bjornstad, O.N., 2020: ncf: Spatial Covariance Functions. https://cran.r-project.org/package=ncf

EDENext, 2014: MODIS v5: Temporal Fourier Analysis (TFA) Imagery Update 2001-12 . Available from: https://www.edenextdata.com/?q=content/modis-v5-temporal-fourier-analysis-tfa-imagery-update-2001-12 [Online].

Elith, J., J.R. Leathwick, and T. Hastie, 2008: A working guide to boosted regression trees. *J. Anim. Ecol.* **77**, 802–813, DOI: 10.1111/j.1365-2656.2008.01390.x.

European Environment Agency, 2018: European Environment Agency, Corine Land Cover 2018 raster data [Online] Available at https://www.eea.europa.eu/data-and-maps/data/copernicus-land-monitoring-service-corine.

Greenwell, B., B. Boehmke, J. Cunningham, and G. Developers, 2019: gbm: Generalized Boosted Regression Models. R package version 2.1.5.

Kjær, L.J., A. Soleng, K.S. Edgar, H.E.H. Lindstedt, K.M. Paulsen, Å.K. Andreassen, L. Korslund, V. Kjelland, A. Slettan, S. Stuen, P. Kjellander, M. Christensson, M. Teräväinen, A. Baum, K. Klitgaard, and R. Bødker, 2019: Predicting the spatial abundance of Ixodes ricinus ticks in southern Scandinavia using environmental and climatic data. Sci. Rep. 9, 18144, DOI: 10.1038/s41598-019-54496-1.

Kuhn., M., T. Contributions from Jed Wing, Steve Weston, Andre Williams, Chris Keefer, Allan Engelhardt, A.Z. Cooper, Zachary Mayer, Brenton Kenkel, the R Core Team, Michael Benesty, Reynald Lescarbeau, and C.C. and T.H. Luca Scrucca, Yuan Tang, 2018: caret: Classification and Regression Training. R package version 6.0-81. https://CRAN.R-project.org/package=caret.

Lunardon, N., G. Menardi, and N. Torelli, 2014: {ROSE}: a {P}ackage for {B}inary {I}mbalanced {L}earning. *{R} J.* **6**, 82–92.

R Development Core Team, 2018: R: A Language and Environment for Statistical Computing. *R Found. Stat. Comput.*http://www.r-project.org. Vienna, Austria: R Foundation for Statistical Computing, Vienna, Austria https://www.R-project.org/.

WorldClim, 2005: WorldClim 1.4 1960-1990 raster data. Available from: http://www.worldclim.org/current [Online].
